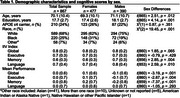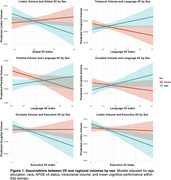# Associations of cognitive intraindividual variability and regional brain volumes vary by sex in the Baltimore Longitudinal Study of Aging

**DOI:** 10.1002/alz70857_104393

**Published:** 2025-12-25

**Authors:** Caitlin M. Terao, Fareshte Erani, Sarah J Banks, Alden L. Gross, Yang An, Keenan A. Walker, Susan M. Resnick, Katherine J. Bangen, Kelsey R. Thomas

**Affiliations:** ^1^ San Diego State University/University of California San Diego Joint Doctoral Program in Clinical Psychology, San Diego, CA, USA; ^2^ University of California, San Diego, La Jolla, CA, USA; ^3^ VA San Diego Healthcare System, San Diego, CA, USA; ^4^ Johns Hopkins Bloomberg School of Public Health, Baltimore, MD, USA; ^5^ Laboratory of Behavioral Neuroscience, National Institute on Aging Intramural Research Program, National Institutes of Health, Baltimore, MD, USA; ^6^ Laboratory of Behavioral Neuroscience, National Institute on Aging, Intramural Research Program, Baltimore, MD, USA

## Abstract

**Background:**

Cognitive intraindividual variability (IIV) is associated with biomarkers of neurodegeneration, long‐term cognitive functioning, and progression to mild cognitive impairment/dementia. However, the extent to which these associations vary by sex are underexplored. The current study examined the differential associations of IIV with brain volumes across male and female older adults.

**Method:**

867 cognitively unimpaired older adults in the Baltimore Longitudinal Study of Aging completed neuropsychological testing and a 3T MRI. Structural neuroimaging markers included total grey and white matter volumes in temporal, parietal, occipital, frontal, and subcortical regions. Global and domain‐specific (executive function, memory, and language) indices of IIV were calculated using intraindividual standard deviation across neuropsychological scores within each construct. Linear regression models examined the associations between IIV and regional brain volume and sex. All models adjusted for intracranial volume, age, education, race, APOE ε4 carrier status, and within‐person mean cognitive performance.

**Result:**

Global, Memory, and Language IIV were not associated with brain volumes across sexes. Among males, greater Global IIV (i.e., more variability) was associated with smaller limbic volumes (β=‐1664.68; 95%CI: ‐3130.18, ‐199.19). In the full sample, greater Executive IIV was associated with lower temporal (β=‐1899.60; 95%CI: ‐3793.67, ‐5.53), limbic (β=‐502.57; 95%CI: ‐954.33, ‐50.80), and subcortical (β=‐797.07; 95%CI: ‐1443.45, ‐150.70) regional volumes. Sex moderated associations between Executive IIV and limbic (β=‐857.64; 95%CI: ‐1776.50, ‐61.23) and occipital regions (β=‐3149.56; 95%CI: ‐6265.53, ‐33.58), such that greater Executive IIV was associated with smaller regional volumes in males but not females. Sex moderated associations between Language IIV and temporal (β=4591.82; 95%CI: 1068.03, 8115.62), parietal (β=3952.94; 95%CI: 426.03, 7479.86), and occipital regions (β=3029.80; 95%CI: 195.14, 5864.47) such that greater Language IIV was associated with smaller regional volumes in females but not males.

**Conclusion:**

In a cognitively healthy sample, Executive IIV was sensitive to temporal, limbic, and subcortical volumes across sexes. Global and Executive IIV related to brain volumes (i.e., limbic and occipital) among older males, whereas Language IIV related to brain volumes (i.e., temporal, parietal, and occipital) among older females. Further work will test these associations longitudinally as well as determine potential sex‐specific mechanisms (e.g., vascular, Alzheimer's disease) for these patterns.